# Effects of outdoor temperature on changes in physiological variables before and after lunch in healthy women

**DOI:** 10.1007/s00484-014-0800-1

**Published:** 2014-03-06

**Authors:** Masahiro Okada, Masayuki Kakehashi

**Affiliations:** 1 Department of Food and Dietetics, Hiroshima Bunka Gakuen Two-Year College, 3-5-1 Nagatsukanishi, Asaminami-ku, Hiroshima, 731-0136 Japan; 2 Graduate School of Biomedical & Health Sciences, Hiroshima University, Hiroshima, Japan

**Keywords:** Physiological variables, Food intake, Outdoor temperature, Salivary amylase activity, Heart rate variability

## Abstract

Previous studies of autonomic nervous system responses before and after eating when controlling patient conditions and room temperature have provided inconsistent results. We hypothesized that several physiological parameters reflecting autonomic activity are affected by outdoor temperature before and after a meal. We measured the following physiological variables before and after a fixed meal in 53 healthy Japanese women: skin temperature, systolic and diastolic blood pressure, salivary amylase, blood glucose, heart rate, and heart rate variability. We assessed satiety before and after lunch using a visual analog scale (100 mm). We recorded outdoor temperature, atmospheric pressure, and relative humidity. Skin temperature rose significantly 1 h after eating (greater in cold weather) (*P* = 0.008). Cold weather markedly influenced changes in diastolic blood pressure before (*P* = 0.017) and after lunch (*P* = 0.013). Fasting salivary amylase activity increased significantly in cold weather but fell significantly after lunch (significantly greater in cold weather) (*P* = 0.007). Salivary amylase was significantly associated with cold weather, low atmospheric pressure, and low relative humidity 30 min after lunch (*P* < 0.05). Cold weather significantly influenced heart rate variability (*P* = 0.001). The decreased low frequency (LF)/high frequency (HF) ratio, increased Δ LF/HF ratio, and increased Δ salivary amylase activity imply that cold outdoor temperature is associated with dominant parasympathetic activity after lunch. Our results clarify the relationship between environmental factors, food intake, and autonomic system and physiological variables, which helps our understanding of homeostasis and metabolism.

**Electronic supplementary material:**

The online version of this article (doi:10.1007/s00484-014-0800-1) contains supplementary material, which is available to authorized users.

## Introduction

Behavior and the environment influence human physiology, with the autonomic nervous system and endocrine axes playing a central role in maintaining homeostasis. There has been significant interest in the role played by the autonomic nervous and endocrine systems in the relationships between outdoor temperature and skin temperature, blood pressure, blood glucose concentration, salivary amylase activity, and heart rate variability. Life requires adaptation to changing outdoor temperatures (LeBlanc 1992; Werner 2008), and the human autonomic nervous system is affected by outdoor temperature (Mäkinen et al. 2008; Mourot et al. 2009). As a result of endocrine responses to the autonomic nervous system, several physiological mechanisms are affected that relate to maintaining homeostasis (Koska et al. 2002; Kanikowska et al. 2013). Eating strongly influences many of these physiological parameters, possibly through similar mechanisms (Harthoorn and Dransfield 2008). The findings of these studies have been inconsistent, and the interplay between outdoor temperature and eating has not been examined.

Exposure to cold provokes a range of physiological responses, predominantly as a consequence of activation of the sympathetic nervous system, including skin temperature, heart rate, diastolic blood pressure, and blood glucose. Skin temperature falls on exposure to cold because of peripheral vasoconstriction (Dériaz et al. 1989; Tappy 1996), although this response may become less intense with repeated exposure (Leppäluoto et al. 2001). For example, the human body adapts to outdoor temperature as winter progresses as a result of thermoregulatory autonomic nervous system adaptations. Ambient temperature also influences heart rate (Banjar et al. 2000), and heart rate variability is used as a physiological index of stress (Mäkinen et al. 2008; van Amelsvoort et al. 2000; Huang et al. 2011). High and uncomfortable ambient temperatures increase sympathetic nervous system activity at the expense of the parasympathetic nervous system, manifested as changes in heart rate variability (Bruce-Low et al. 2006; Liu et al. 2008). Diastolic blood pressure tends to rise in cold weather (Jehn et al. 2002; Halonen et al. 2011), and blood glucose concentration appears to be higher in winter than summer (Behall et al. 1984; Tseng et al. 2005; Kershenbaum et al. 2011). Sympathetic nervous system activity and hormones, such as noradrenaline, are stimulated by cold stress and lead to vasoconstriction and increased plasma glucose concentration. Outdoor temperature appears to influence several aspects of metabolism, including resting metabolic rate and brown adipose tissue, even when measurements are taken in comfortable indoor conditions (Kashiwazaki et al. 1990; Nedergaard et al. 2007). Indeed, fluctuations in outdoor temperature are postulated to be the best explanation for seasonal variations in blood pressure (Sinha et al. 2010; Kanikowska et al. 2013).

Eating also induces a number of important physiological changes. Central body temperature rises after a meal as a result of increased metabolic activity by the liver (Westerterp-Plantenga et al. 1990). However, skin temperature is not thought to change after eating. Blood pressure tends to fall after meals, judged to be a consequence of dominant parasympathetic activity in the autonomic nervous system (Fagan et al. 1986; Peitzman and Berger 1989). The activity of salivary amylase, an α-amylase that initiates digestion of starches, is increased by eating but also appears to be stimulated by the sympathetic nervous system, although the mechanism is still not fully understood (Nater and Rohleder 2009; Bosch et al. 2011).

Many of these factors play a role in the regulation of appetite and satiety, although other factors, including appetite-regulating gut hormones, also play a crucial role (Wasse et al. 2013). For example, heart rate variability changes in a way that suggests sympathetic nervous system predominance until an hour after eating, when the balance shifts in favor of the parasympathetic nervous system (Lu et al. 1999; Harthoorn and Dransfield 2008). There appears to be an association between heart rate variability and obesity (Matsumoto et al. 1999; Tentolouris et al. 2003; Fujibayashi et al. 2009; Paschoal et al. 2009), and salivary amylase activity is positively correlated with satiety (Harthoorn and Dransfield. 2008).

However, the influences of outdoor temperature and eating on these physiological variables and satiety have rarely been examined together. To observe the influence of outdoor temperature on satiety and the physiological changes brought about by eating, we measured heart rate, heart rate variability, blood pressure, axillary temperature, and salivary amylase activity before and after lunch in a cohort of healthy young Japanese women and sought correlations with outdoor temperature, relative humidity, and atmospheric pressure.

## Participants and methods

### Participants and study design

Fifty-three healthy female university students aged 18–29 years volunteered to participate in this study, which was approved by the Human Studies Committee of Hiroshima Bunka Gakuen Two-Year College. Informed consent was obtained from all participants. We studied females because previous studies on physiological responses to eating or temperature were conducted using female participants (Matsumoto et al. 1999; 2001; Fujibayashi et al. 2009; Tentolouris et al. 2003). The study was performed between March 2010 and March 2012. Nineteen of the recordings were made in autumn and winter (36 %) and 34 in spring and summer (64 %). Each participant completed the study in 1 day on either a holiday or nonworking day. One participant was studied per day, and no participant participated twice. We selected nonsmokers, who were not taking prescription medication, and had no history of cardiovascular or endocrine disease. The exclusion criteria were excessive weight loss during the last 3 months, a body mass index >30 kg/m^2^, and menstruation during the study. All participants verbally described their breakfast meal contents and confirmed that they had not consumed alcohol, caffeine, or capsaicin. We also interviewed all participants and confirmed that they were in good health, getting adequate sleep, and had fasted since breakfast. Weight and height were measured while the participants wore light indoor clothing, with empty pockets, and no shoes. Body fat proportion was measured using a body composition meter (model BC-520; Tanita Corporation, Tokyo, Japan). We limited the amount of exercise before lunch to avoid the effects of strenuous exercise on body fat measurement. We did not limit water intake prior to the measurements but did adjust for body fat percentage in the multiple regression analysis. Satiety was assessed using a visual analog scale (100 mm) before and after lunch.

Room temperature was maintained within 20–25 °C (Kashiwazaki et al. 1990). Measurements were taken after the participant had adapted to the temperature of the room for 1 h, wearing light indoor clothes (underwear and a long-sleeved T-shirt). Data were collected for each individual on four occasions: before lunch, immediately after lunch, 30 min after lunch, and 1 h after lunch. The participants had refrained from eating snacks or engaging in physical activity since breakfast. All participants ate the same lunch (total energy 806 kcal; 66.4 % carbohydrates, 12.8 % protein, 20.8 % fat; no caffeine or capsaicin; energy index, 40.3 % of total daily requirement, based on an estimate of 2,000 kcal/day). The mean time taken to eat lunch was 16.2 ± 8.2 min (mean ± standard deviation (SD); range, 7–43 min) and there was no time limit.

The mean outdoor temperature, atmospheric pressure, and relative humidity values, listed in Table 1, were obtained from the Hiroshima Local Meteorological Observatory (3 km from our laboratory) between 11:30 a.m. and 1:30 p.m.

**Table 1:** Participant characteristics and outdoor environmental conditions (*n* = 53)

	Mean ± SD	Range
Participant characteristics		
Age (years)	20.4 ± 2.6	18–29
Body mass index (kg/m^2^)	20.7 ± 2.7	16.6–29.2
Proportion of body fat (%)	28.6 ± 5.1	17.4–40.9
Environmental conditions		
Outdoor temperature (°C)	18.0 ± 9.2	1.3–31.6
Atmospheric pressure (hPa)	1,007.5 ± 6.5	994.7–1,022.4
Relative humidity (%)	66.2 ± 10.0	41.0 to 89.0

### Measurement of physiological variables

Recordings were made with the participant in a quiet, well-ventilated, and well-lit room between 11:30 a.m. and 1:30 p.m. Baseline measurements were taken ≥3 h after the participant had eaten breakfast. Skin temperature was measured in the axilla (four readings, Omron MC-170 fully automatic monitor, Omron Healthcare Corporation, Kyoto, Japan), allowing 10 min for equilibrium to be reached. Axillary temperature was chosen rather than measuring skin temperature over the region of the liver because the axillary method is the most commonly used method in Japan and because we felt that we would obtain greater participant consent using this method, given that we took four readings. Noninvasive systolic and diastolic blood pressures were measured in the upper arm using a cuff and an Omron HEM-1000 fully automatic monitor (Omron Healthcare Corporation). Blood glucose concentration was determined by the glucose oxidase method (Sanwa Kagaku Kenkyusho Corporation, Nagoya, Japan) from finger prick samples. Salivary amylase activity (fasting and after lunch) was measured using a hand-held noninvasive monitor (Nipro Corporation, Osaka, Japan) based on the 2-chloro-4-nitrophenyl-4-galactopyranosylmaltoside hydrolysis method (Yamaguchi et al. 2006).

### Heart rate and heart rate variability

Heart rate and heart rate variability were determined from individual datasets recorded using an SA-3000P device (Tokyo Iken Corporation, Tokyo, Japan). Power spectral analysis of heart rate variability was used to determine the R-R interval, and spectrum estimation was analyzed by fast Fourier transformation. Heart rate variability analysis was carried out based on the guidelines of the Task Force of the European Society of Cardiology and the North American Society of Pacing and Electrophysiology (1996) in spontaneously breathing patients, ensuring the reproducibility of our results (Dionne et al. 2002). Spectral analysis was split into high frequency (HF, 0.15–0.40 Hz) and low frequency (LF, 0.04–0.15 Hz) components. The HF component reflects parasympathetic nervous system activity, whereas the LF component reflects sympathetic nervous system activity and some parasympathetic activity. We calculated the LF/HF ratio, which reflects the balance between the activities of the sympathetic and parasympathetic nervous systems (Akselrod et al. 1981; Pagani et al. 1986).

### Data analysis

All statistical analyses were performed using SPSS software (version 17.0, IBM SPSS Inc., Tokyo, Japan). Data for all participants and outdoor environmental conditions are expressed as means ± SD or standard error (SE). The distribution of data was initially examined using the Shapiro–Wilk test then analyzed using parametric or nonparametric tests, accordingly. The Friedman test was performed to assess temporal changes in physiological variables before and after lunch. The Wilcoxon-signed rank test was performed to compare physiological variables recorded before lunch (baseline) with each time point after lunch. Multiple linear regression analyses were undertaken to determine associations between changes in physiological variables from the baseline levels recorded before lunch and outdoor environmental conditions. These analyses were performed after adjusting for room temperature, age, body mass index, and proportion of body fat. To further examine the relationships between salivary amylase activity, LF/HF ratio, and outdoor temperature, unpaired *t* tests were used to compare fasting salivary amylase activity or LF/HF ratio 1 h after lunch in three groups of participants stratified by outdoor temperature (low, < 10.0 °C; mid, 10.0–20.0 °C; high, >20.0 °C). Values of *P* < 0.05 were considered statistically significant. Changes in satiety before and after lunch were analyzed using the Friedman test and the Wilcoxon-signed rank test. Satiety before and after lunch was assessed by stratifying by outdoor temperature as for LF/HF ratio.

## Results

### Participant characteristics

Participant characteristics are shown in Table 1. Thirteen participants declined to give saliva samples and 19 declined to give blood; therefore, salivary amylase activity was measured in 40 participants and blood glucose concentration was measured in 34 participants.

### Influence of eating on physiological variables

Diastolic blood pressure and salivary amylase activity both fell significantly after lunch compared with baseline. Systolic blood pressure did not change significantly. Blood glucose concentration and heart rate rose significantly at all time points after eating compared with baseline. Skin temperature also rose after eating, but this was significantly higher than baseline only at 1 h. LF/HF ratio also fell after eating, indicating the predominance of parasympathetic activity; however, this was statistically significant only at 1 h after lunch (for all results, see Table 2).

**Table 2:** Changes in physiological variables before and after lunch

Physiological variables	*n*	Immediately before lunch	Immediately after lunch	30 min after lunch	1 h after lunch	Significance
Skin temperature (°C)	53	35.8 ± 0.5	35.9 ± 0.6	36.0 ± 0.5	36.0 ± 0.5*	ns
Systolic blood pressure (mmHg)	53	105.6 ± 10.1	103.0 ± 10.0	104.3 ± 10.1	103.8 ± 14.0	ns
Diastolic blood pressure (mmHg)	53	63.1 ± 8.1	58.4 ± 7.4**	58.9 ± 10.0**	61.2 ± 13.3**	*P* < 0.01
Salivary amylase activity (kU/L)	40	78.2 ± 65.7	62.2 ± 62.4*	54.2 ± 42.2**	65.4 ± 55.4*	*P* < 0.01
Plasma glucose levels (mmol/L)	34	5.0 ± 0.7	7.2 ± 0.9**	7.6 ± 1.3**	6.6 ± 1.3**	*P* < 0.01
Heart rate (beats/min)	53	73.1 ± 10.2	77.6 ± 11.3**	78.1 ± 12.2**	76.2 ± 11.8**	*P* < 0.01
Low-to-high frequency ratio	53	1.8 ± 1.8	1.8 ± 2.3	1.5 ± 1.5	1.3 ± 1.3**	*P* < 0.01

Values are means ± standard deviation. Overall *P* values were determined using the Friedman test

*ns* not significant

**P < 0.01, *P < 0.05 vs. before lunch (Wilcoxon-signed rank test)

### Influence of outdoor temperature on physiological findings before lunch

**Fig. 1:**
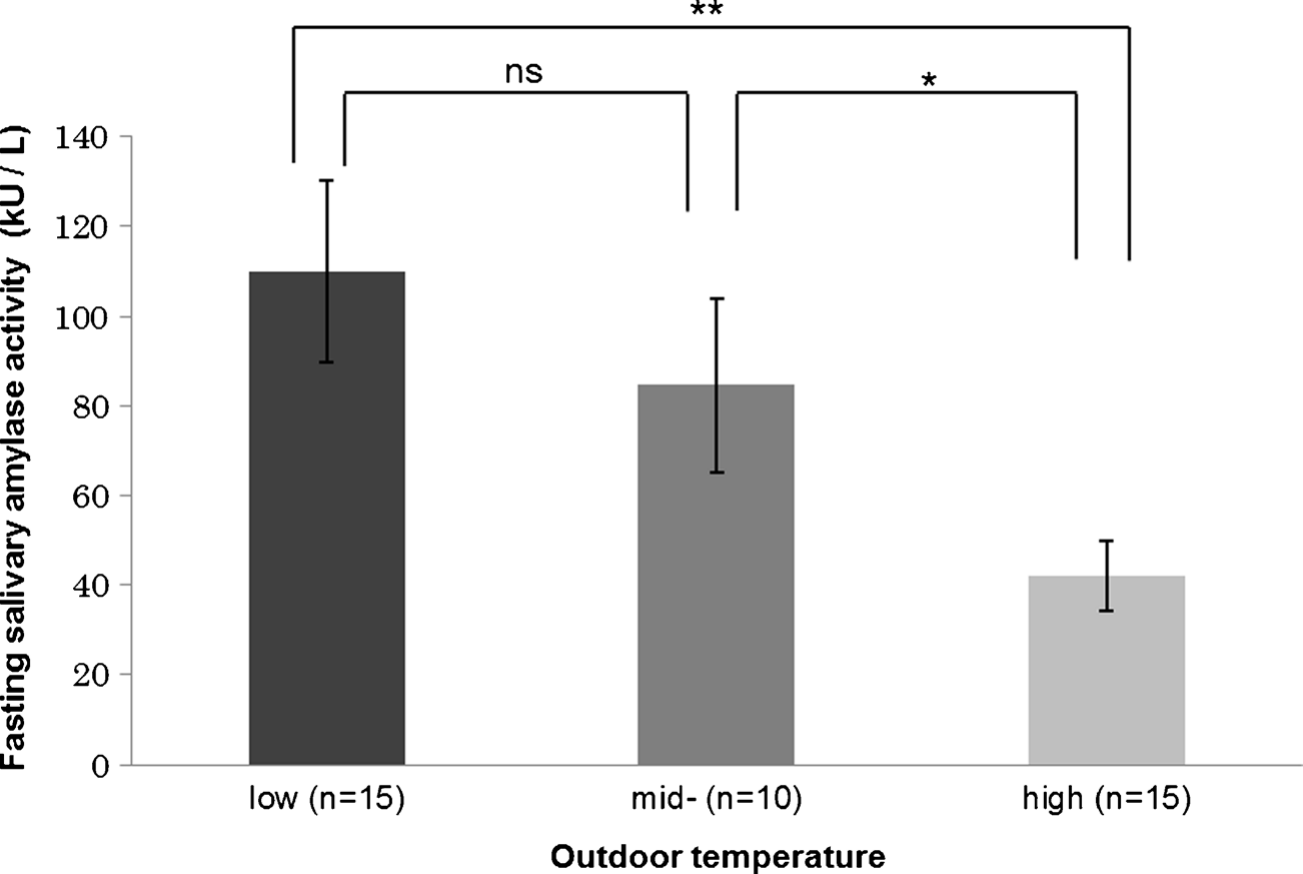
Fasting salivary amylase activity stratified by outdoor temperature (low <10.0 °C; mid 10.0–20.0 °C; high >20.0 °C). Values are means ± standard error. *ns* not significant *P < 0.05, **P < 0.01

Before eating, we found that low outdoor temperature was significantly associated with increased diastolic blood pressure, increased fasting salivary amylase activity, and increased fasting blood glucose concentration (Table 3). When outdoor temperature was subdivided into low (<10.0 °C), mid (10.0–20.0 °C), and high (>20.0 °C) groups, participants studied during hotter conditions had significantly lower fasting salivary amylase activity (Fig. 1). We found no significant relationships between outdoor temperature and skin temperature, systolic blood pressure, heart rate, or heart rate variability. Relatively lower atmospheric pressure was significantly associated with increased skin temperature, but otherwise neither atmospheric pressure nor relative humidity appeared to have any influence on the physiological parameters recorded (Table 3).

**Table 3:** Relationships between physiological variables and outdoor environmental conditions

Variables	Conditions	Immediately before lunch *β* (*P*)	Immediately after lunch *β* (*P*)	30 min after lunch *β* (*P*)	1 h after lunch *β* (*P*)
Skin temperature *n* = 53	Outdoor temperature	−0.048 (0.774)	−0.184 (0.297)	−0.306 (0.067)	−0.400 (0.014)
	Atmospheric pressure	−0.382 (0.031)	−0.365 (0.046)	−0.314 (0.066)	−0.366 (0.027)
	Relative humidity	0.127 (0.383)	−0.006 (0.971)	0.164 (0.250)	0.164 (0.223)
Systolic blood pressure *n* = 53	Outdoor temperature	−0.212 (0.228)	−0.301 (0.081)	−0.341 (0.045)	−0.293 (0.060)
	Atmospheric pressure	−0.019 (0.917)	−0.067 (0.701)	−0.093 (0.587)	−0.049 (0.756)
	Relative humidity	0.057 (0.738)	0.009 (0.952)	0.130 (0.570)	0.031 (0.812)
Diastolic blood pressure *n* = 53	Outdoor temperature	−0.408 (0.017)	−0.401 (0.013)	−0.271 (0.063)	−0.199 (0.145)
	Atmospheric pressure	0.056 (0.741)	−0.057 (0.719)	−0.062 (0.670)	0.076 (0.581)
	Relative humidity	−0.048 (0.738)	−0.246 (0.073)	−0.020 (0.869)	−0.044 (0.706)
Salivary amylase activity *n* = 40	Outdoor temperature	−0.463 (0.017)	−0.263 (0.220)	0.031 (0.882)	0.177 (0.446)
	Atmospheric pressure	0.025 (0.897)	0.064 (0.772)	0.383 (0.085)	0.153 (0.522)
	Relative humidity	−0.147 (0.366)	−0.159 (0.392)	0.187 (0.303)	0.035 (0.857)
Plasma glucose levels *n* = 34	Outdoor temperature	−0.600 (0.003)	−0.305 (0.158)	0.172 (0.458)	−0.219 (0.296)
	Atmospheric pressure	−0.079 (0.678)	0.022 (0.918)	0.001 (0.997)	−0.304 (0.154)
	Relative humidity	0.240 (0.148)	0.258 (0.170)	−0.235 (0.248)	−0.153 (0.397)
Heart rate *n* = 53	Outdoor temperature	−0.019 (0.909)	−0.091 (0.608)	−0.076 (0.666)	0.056 (0.749)
	Atmospheric pressure	−0.046 (0.785)	−0.052 (0.773)	0.042 (0.818)	0.168 (0.349)
	Relative humidity	0.023 (0.870)	0.057 (0.710)	0.034 (0.826)	0.073 (0.628)
LF/HF ratio *n* = 53	Outdoor temperature	0.247 (0.143)	0.254 (0.158)	0.254 (0.149)	0.587 (<0.001)
	Atmospheric pressure	0.102 (0.344)	0.031 (0.866)	0.128 (0.474)	0.384 (0.019)
	Relative humidity	0.080 (0.580)	0.062 (0.688)	−0.187 (0.216)	0.108 (0.423)

Analysis was made after adjusting for room temperature, age, body mass index, and proportion of body fat. *P* values are shown in parentheses

*β* standard regression coefficient, *LF/HF* low-to-high frequency ratio

### Influence of outdoor temperature on physiological findings after lunch

**Fig. 2:**
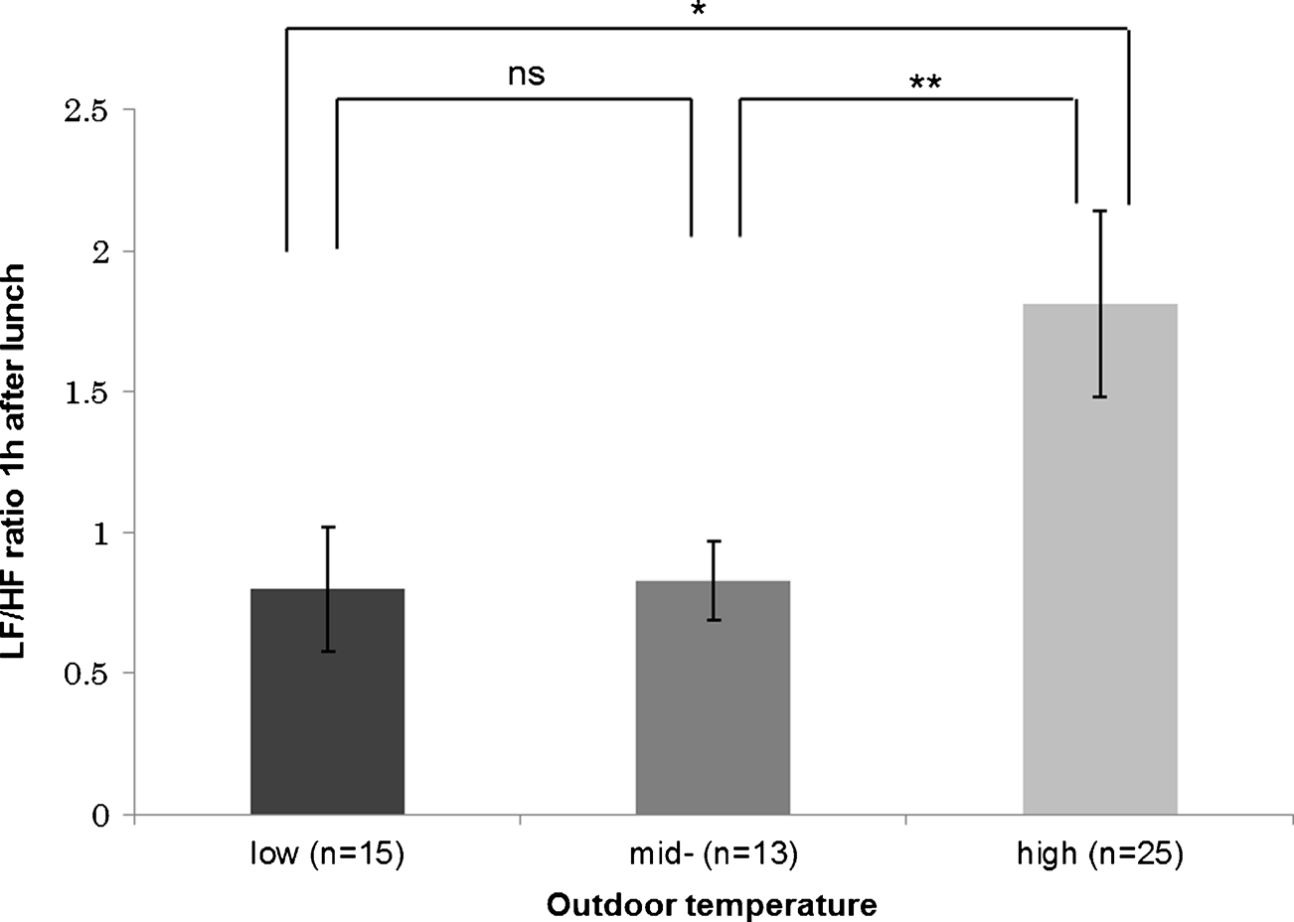
LF/HF ratio 1 h after lunch stratified by outdoor temperature (low <10.0 °C, mid 10.0–20.0 °C; high >20.0 °C). Values are means ± standard error. *ns* not significant, *P < 0.05, **P < 0.01

Cold outdoor conditions were significantly associated with increased skin temperature 1 h after lunch, increased systolic blood pressure 30 min after lunch, increased diastolic blood pressure immediately after lunch, and a decreased LF/HF ratio 1 h after lunch (Table 3). Hotter outdoor temperatures resulted in a higher mean LF/HF ratio 1 h after lunch (Fig. 2). Lower atmospheric pressure was associated with significantly increased skin temperature immediately and 1 h after lunch and decreased LF/HF ratio 1 h after lunch. Relative humidity did not influence any of the variables we measured at any time.

We found significant relationships between environmental conditions and change in skin temperature between baseline and 1 h after lunch (Δ skin temperature), salivary amylase activity (Δ salivary amylase activity), and LF/HF ratio (Δ LF/HF ratio) (Table 4), but not between any of the other physiological variables (data not shown). As well as reflecting the associations described above, this analysis also revealed that cold weather was associated with significant negative changes from baseline in skin temperature 1 h after lunch, salivary amylase activity at 30 min and 1 h, and LF/HF ratio at 1 h. Atmospheric pressure and relative humidity had a similarly significant influence on salivary amylase activity 30 min after eating. Lower atmospheric pressure was associated with a significant negative change in LF/HF ratio 1 h after eating (Table 4).

**Table 4:** Relationships between outdoor environmental conditions and changes in physiological variables after lunch

Variables	Conditions	Immediately after lunch *β* (*P*)	30 min after lunch *β* (*P*)	1 h after lunch *β* (*P*)
Δ Skin temperature *n* = 53	Outdoor temperature	−0.176 (0.295)	−0.291 (0.056)	−0.384 (0.008)
	Atmospheric pressure	−0.165 (0.360)	−0.137 (0.397)	−0.184 (0.224)
	Relative humidity	−0.008 (0.543)	0.106 (0.413)	−0.107 (0.377)
Δ Salivary amylase activity *n* = 40	Outdoor temperature	−0.099 (0.647)	−0.333 (0.016)	−0.490 (0.007)
	Atmospheric pressure	−0.055 (0.789)	−0.299 (0.021)	−0.124 (0.445)
	Relative humidity	0.059 (0.732)	−0.250 (0.022)	−0.135 (0.320)
Δ LF/HF ratio *n* = 53	Outdoor temperature	0.112 (0.517)	−0.137 (0.339)	−0.422 (0.001)
	Atmospheric pressure	−0.086 (0.623)	−0.057 (0.693)	−0.276 (0.034)
	Relative humidity	−0.012 (0.932)	0.184 (0.131)	−0.064 (0.545)

Analysis was undertaken on the change from baseline recordings of each parameter made before lunch. *P* values are shown in parentheses

*β* standard regression coefficient, *LF/HF* low-to-high frequency ratio

### Influence of outdoor temperature on satiety findings before and after lunch

Satiety peaked immediately after lunch and fell gradually thereafter (Online Resource 1). When outdoor temperature was stratified, satiety with low temperature (<10 °C) was higher than with mid (10–20 °C) or high temperature (>20 °C) before lunch, but after eating, higher satiety occurred with high outdoor temperature than with mid or low temperature (Online Resource 2). However, we found no significant relationships between physiological variables and satiety (data not shown).

## Discussion

We found that colder outdoor temperatures were associated with increased diastolic blood pressure, increased salivary amylase activity, and increased blood glucose concentration in fasting healthy volunteers. Cold outdoor temperatures also appeared to influence the autonomic, and possibly endocrine, response to eating. At various times after eating, cold outdoor conditions were associated with increased skin temperature, increased systolic and diastolic blood pressures, decreased salivary amylase activity, and a decreased LF/HF ratio.

Although it has previously been reported that liver temperature increases after eating but that skin temperature does not (Westerterp-Plantenga et al. 1990), we found a slight increase in skin temperature 1 h after lunch, likely because of peripheral vasodilation. Interestingly, the increase in skin temperature after eating was more marked if it was cold outside or there was low atmospheric pressure, with temperature appearing to have the strongest influence. When there is a large difference between skin temperature and outdoor temperature after lunch, heat energy expenditure of the body increases (van Marken Lichtenbelt et al. 2001) and Δ skin temperature increases. Also, because parasympathetic nervous system activity and insulin effect changes in body temperature (Dériaz et al. 1989; Tappy 1996), our results suggest that the parasympathetic nervous system is more strongly activated after lunch when it is cold outside.

We found a significant increase in diastolic blood pressure in cold weather before eating and a significant decrease after eating, which is expected in young women (Fagan et al. 1986; Peitzman et al. 1989). In cold weather, the change in diastolic blood pressure was particularly marked immediately after lunch, but outdoor temperature had no apparent influence at 30 min or 1 h. Systolic blood pressure did not change significantly after lunch but was significantly inversely associated with outdoor temperature 30 min after eating. Our findings suggest that immediately after eating, diastolic blood pressure is more susceptible to the effects of food intake and outdoor temperature than systolic blood pressure and that peripheral vasodilation after food intake might mask any effects of outdoor temperature on diastolic blood pressure 30 min after eating. Blood pressure is influenced by outdoor temperature and seasonality (Hayashi et al. 2008; Morabito et al. 2008; Kamezaki et al. 2010; Sinha et al. 2010), although the underlying mechanism has not been explained and there have been conflicting results as to whether systolic pressure (Sinha et al. 2010) is more affected than diastolic pressure (Halonen et al. 2011). We judge that low outdoor temperatures increase baseline blood pressure and that the autonomic and endocrine responses to eating override the influence of outdoor temperature.

We found that fasting salivary amylase activity was associated with decreased outdoor temperatures. Bhattacharyya and Datta (1986) found a relationship between salivary amylase activity and decreased temperature, but no human studies have shown this link. In humans, O’Donnell et al. (2009) studied the relationship between salivary amylase and the cold hand test, but found no correlation. Previous reports showed that salivary amylase activity increases in response to food intake or satiety, suggesting increased sympathetic nervous system tone (Harthoorn and Dransfield 2008). Unexpectedly, we found a significant decrease in salivary amylase activity after lunch, which was also strongly influenced by cold outdoor temperature at 30 min and 1 h and low atmospheric pressure and relative humidity at 30 min. Our findings imply that in our participants, the parasympathetic nervous system became dominant after lunch, particularly in cold weather.

Our participants’ fasting blood glucose concentration tended to be significantly higher when it was cold outside, which concurs with previous findings (Kamezaki et al. 2010; Kershenbaum et al. 2011). This fits with the concept of environmental stress altering autonomic and endocrine responses; however, unlike salivary amylase activity, we did not find any associations between postprandial blood glucose concentration and outdoor environmental conditions, and we did not measure insulin levels to be able to draw conclusions. The outdoor environmental conditions experienced by our participants may not have been extreme enough to alter the homeostasis of glucose metabolism.

It is well recognized that heart rate increases after food intake (Harthoorn and Dransfield 2008), but the rise we observed was not influenced by outdoor environmental conditions, possibly because these were not particularly extreme (Rodahl 2003). With respect to heart rate variability, we found a marked decrease in the LF/HF ratio 1 h after lunch, supporting the concept that at least a proportion of the associations we found can be explained by changes in autonomic nervous system activity.

Previous studies of heart rate variability have reported conflicting findings: Harthoorn et al. (2008) reported that the LF/HF ratio decreased 1 h after food intake, but Lu et al. (1999) reported the opposite. The decrease in LF/HF ratio 1 h after eating reflects parasympathetic dominance at that time, and this was even more pronounced in our study in cold weather or at low atmospheric pressure. The LF/HF ratio is increased by exposure to uncomfortably high ambient temperatures (Bruce-Low et al. 2006; Liu et al. 2008), and we propose that the influence of outdoor environmental factors on the autonomic nervous system is different in fasted and fed states. We also speculate that lower outdoor temperature and atmospheric pressure further increase the dominance of the parasympathetic nervous system 1 h after eating, resulting in decreasing salivary amylase activity and increasing skin temperature (perhaps by further stimulating hepatic metabolism, inhibiting peripheral vasoconstriction, or both).

Although we found no significant relationships between satiety and outdoor temperature, we did find that satiety tended to be higher with low temperature before lunch and with high temperature after lunch. Although we found no relationships between physiological variables and satiety, Harthoorn and Dransfield (2008) found a positive correlation with salivary amylase activity and satiety. We found a strong decrease in salivary amylase activity after lunch with low outdoor temperature. Based on these results, we suggest that low outdoor temperature reduces satiety after eating. In cold weather, if satiety decreases after eating, appetite may increase and we suggest that physiological changes may be related to satiety after eating, including a relationship with energy intake and ghrelin levels (Wasse et al. 2013).

Our findings suggest that outdoor temperature affects the physiological variables before and after lunch in the following order (from strongest to weakest): heart rate variability, salivary amylase activity, skin temperature, diastolic blood pressure, plasma glucose concentration, systolic blood pressure, and heart rate.

Our study had several limitations. First, we chose to study young healthy women living in Hiroshima, as women’s sensitivity to climate stress appears to differ from that of men (Bortkiewicz et al. 2006), so findings may not be generalizable to other populations. Second, we did not take into account the relationship with daylight length, which is known to alter physiological responses (Shephard and Aoyagi 2009). Diurnal rhythms have a steady background influence on all of the physiological variables that we measured. This background influence might be affected by behavior and eating habits, even without changes in temperature or pressure. In addition to diurnal rhythms, heart rate variability might also be affected by other factors such as particulate matter (Styer et al. 1995; Creason et al. 2001). Third, we examined physiological changes only up to 1 h after eating, so we are unable to comment on whether there might be longer term influences. Fourth, if participants were left to consume an unlimited quantity of food, the influence of outdoor temperature may lead to different changes in physiological variables after a meal. Westerterp-Plantenga et al. (2002) showed that overeating under ad libitum circumstances at 16 °C attenuated the decrease in rectal core body temperature. Stimulation by overeating may lead to sympathetic nervous system dominance, strongly affect satiety, and may strongly influence changes in physiological variables and energy balance. Fifth, we had an unequal number of participants in the two seasons purely by chance as a result of our participants volunteering for one data collection session or the second. However, because our main focus was on finding relationships between outdoor temperature and the measured variables, and not seasonality, the different proportions in the two groups were not a factor. Finally, our study was observational, so we were not able to pinpoint the mechanisms that underpin the physiological changes that we report. Despite these limitations, we found new relationships between outdoor temperature and the physiological changes associated with eating and evidence that suggests that the autonomic nervous system plays at least a part in the underlying mechanisms.

In conclusion, body temperature, salivary amylase activity, and heart rate variability after lunch are strongly affected by outdoor temperature, as a result of autonomic activity and endocrine factors. We speculate that these are normal homeostatic mechanisms that are essential for the maintenance of health and that are likely to affect endogenous adrenergic activity and many hormones, including cortisol, thyroid hormones, leptin, and ghrelin. This area requires further study.

In cold weather, the greater increase in autonomic activity after eating appears to reduce satiety and might stimulate appetite in cold or stressful conditions. This relationship may explain the variation in eating habits in different regions and during different seasons. The results from our study help determine healthy food intakes in different environments by providing preliminary baseline data for future studies. A better understanding of the relationship between environmental factors, food intake, and autonomic and endocrine activities improves our understanding of homeostasis and metabolism.

## Electronic supplementary material

Below is the link to the electronic supplementary material.

ESM 1 (PDF 304 kb)
