# Supplementary material for: Effects of outdoor temperature on changes in physiological variables before and after lunch in healthy women
Source: Int J Biometeorol. 2014 Mar 6;58(9):1973–81. doi: 10.1007/s00484-014-0800-1 (PMC4190455; doi:10.1007/s00484-014-0800-1)
Supplement: Supplementary file 1 — (PDF 304 kb) [file 484_2014_800_MOESM1_ESM.pdf]

**Supplementary Data**

**Effects of outdoor temperature on changes in physiological variables before and after lunch in healthy women**

***International Journal of Biometeorology***

Masahiro Okada • Masayuki Kakehashi

Masahiro Okada (🖂)
Department of Food and Dietetics, Hiroshima Bunka Gakuen Two-Year College, 3-5-1 Nagatsukanishi, Asaminami-ku, Hiroshima 731-0136, Japan
E-mail: okada@hbg.ac.jp

Satiety data

**Online Resource 1** Change in satiety variables before and after lunch based on VAS data

| Variables | n | Immediately before lunch | Immediately after lunch | 30 min after lunch | 1 h after lunch | Significance |
|---|---|---|---|---|---|---|
| Satiety level (mm) | 53 | 44.6±1.7 | 89.5±0.8 ** | 78.8±1.1 ** | 72.8±1.4 ** | $p<0.01$ |

Satiety variables were measured by 100 mm visual analogue scales in all 53 participants.

Values are means ± standard deviation.

$P$ values were determined using the Friedman test.

**$P<0.01$ vs. before lunch (Wilcoxon-signed rank test).

**Online Resource 2** Satiety variables before and after lunch stratified by outdoor temperature

| Variables | Outdoor temperature | n | Immediately before lunch | Immediately after lunch | 30 min after lunch | 1 h after lunch |
|---|---|---|---|---|---|---|
| Satiety (mm) | high: >20.0°C | 25 | 42.9±3.4 | 91.6±1.5 | 80.4±2.3 | 73.9±3.0 |
| | middle: 10.0–20.0°C | 13 | 40.9±4.3 | 87.6±2.4 | 77.4±2.7 | 70.8±4.3 |
| | low: <10.0°C | 15 | 50.9±4.3 | 87.7±1.8 | 77.6±3.2 | 72.7±3.0 |

Values are means ± standard error. Satiety variables were measured by 100 mm visual analogue scales in all 53 participants.
